# 

**Published:** 1883-02

**Authors:** 


Vol. XXXVIII. FEBRUARY, 1883. No. 2.

### THE

### Dental Register.

### A MONTHLY JOURNAL,

DEVOTED TO THE INTERESTS OF THE PROFESSION.

EDITED BY J. TAFT, D.D.S.

PUBLISHED BY

SPENCER & CROCKER,

OHIO DENTAL DEPOT,

Nos. 117, 119, 121 WEST FIFTH STREET, CINCINNATI, OHIO.

**TERMS:—$2.00 Per Annum, in Advance. Single Copies, 25 cts.**

